# Intratumoral bacterial load and tertiary lymphoid structure density in hepatocellular carcinoma: association and prognostic significance

**DOI:** 10.3389/fimmu.2025.1652433

**Published:** 2025-09-02

**Authors:** Shen Qu, Weili Jia, Xiaoyan Liu, Qianyun Yao, Chao Chen, Zihao Zhao, Ye Nie, Feng Chang, Zexu Yang, Chaosheng Peng, Yangang Wang, Wenjie Song

**Affiliations:** ^1^ Department of Hepatobiliary Surgery, The First Affiliated Hospital of Air Force Military Medical University, Xi’an, China; ^2^ Xi’an Medical University, Xi’an, China; ^3^ Lanzhou University, Lanzhou, Gansu, China; ^4^ Department of Geriatrics, The First Affiliated Hospital of Air Force Military Medical University, Xi’an, China

**Keywords:** hepatocellular carcinoma, intratumoral bacterial, tertiary lymphoid structure, tumor immune microenvironment, microbiota

## Abstract

**Background:**

The tertiary lymphoid structures (TLS) within the immune microenvironment of hepatocellular carcinoma (HCC) have been shown to significantly influence patient prognosis. Understanding the mechanisms behind their formation and maturation can help us refine therapeutic strategies and improve treatment outcomes. In certain cancers, intratumoral bacteria have been found to promote the development of TLS. Our study aims to investigate the impact of intratumoral bacteria on TLS-associated immune cells in HCC, as well as their relationship with TLS quantity and maturation status.

**Methods:**

In this study, we collected samples from 153 patients with hepatocellular carcinoma. We employed fluorescence *in situ* hybridization (FISH), immunofluorescence (IF), and hematoxylin and eosin (H&E) staining to assess intratumoral bacterial load, as well as to evaluate the number and maturation status of tertiary lymphoid structures. Patient prognosis was also analyzed. Furthermore, we examined the bacterial load and distribution within tumors of patients presenting TLS, and explored the relationship between intratumoral bacteria and TLS-associated immune cell infiltration.

**Results:**

Among 74 patients with hepatocellular carcinoma, tertiary lymphoid structures were present within the tumors. By integrating the expression profiles of both intratumoral and peritumoral TLS, prognostic analysis revealed that patients with structured tumor microenvironments (TME) and exclusionary TME had better outcomes. The intratumoral bacterial load varied among patients, with higher bacterial burden observed in regions enriched with TLS. Moreover, as TLS matured, the bacterial load within tumors was significantly greater compared to patients lacking TLS. Correspondingly, CD20, a major component of TLS, showed increased expression. These findings suggest that intratumoral bacteria can influence the immune response within the tumor microenvironment and are associated with the maturation of TLS.

**Conclusion:**

Our study demonstrates that patients receiving structured TME and excluded TME subtypes exhibit superior overall survival (OS) and recurrence-free survival (RFS) following radical surgery. Furthermore, the intratumoral bacterial load showed a significant correlation with CD20+ B-cell density and was strongly correlated with both the number and maturity of TLS. These findings suggest that intratumoral bacteria may influence patient responses to immunotherapy.

## 1 Introduction

Primary liver cancer is the third leading cause of cancer deaths globally (1, 2), It mainly includes hepatocellular carcinoma, cholangiocarcinoma, etc., especially hepatocellular carcinoma, which is mainly caused by liver cirrhosis or nonalcoholic steatohepatitis (3, 4). Despite the availability of various treatments, including surgical resection and immunotherapy” or “such as surgical resection and immunotherapy, surgical resection being the most prevalent, most patients experience poor five-year survival rates, and prone to recurrence (5). Postoperative combination immunotherapy has emerged as a primary treatment strategy for various cancers in recent years. Nevertheless, its effectiveness frequently remains limited in HCC (6), Therefore, we need to have a deeper understanding of the tumor immune microenvironment of HCC in order to provide better treatment for patients.

Research has identified the presence of TLS within tumor tissues. These organized aggregates of immune cells are primarily formed through the recruitment of B cells, T cells, dendritic cells (DCs), and other immune cells by chemokines or other factors. Among these components, B cells constitute the predominant cellular population within TLS (7). B cells are also a major component of the tumor microenvironment in many types of cancer (8). The maturation markers of tertiary lymphoid structures, CD21 and CD23, are both expressed by B cells. CD21 is primarily expressed on follicular dendritic cells and B lymphocytes. CD23, a differentiation antigen of B lymphocytes, is also expressed by these cells (8). Both markers serve to visualize the follicular dendritic cells network within germinal centers, which plays a crucial role in regulating B lymphocyte activation. The antitumor function of TLS formed within human tumors is primarily dependent on antibody-mediated humoral immune responses driven by B lymphocyte-secreted antibodies (7, 9). Prognostic studies in diverse cancers consistently report that the presence of TLS is associated with superior objective response rates to immunotherapy and better clinical outcomes when compared to TLS-negative tumors (10–12), At present, we know very little about the formation mechanism of TLS.

Recent years have witnessed increasing research interest in bacteria residing within tumors (13), It has been proposed in report that intratumoral bacteria might contribute to the development of tertiary lymphoid structures. Bacterial colonization and persistent growth occur within tumors (14), In cancers such as breast cancer (15) and colorectal cancer (16), these intratumoral bacteria primarily reside intracellularly within cancer cells (17), infection with bacteria leads to the upregulation of pro-oncogenic genes in tumor cells (18), enhancing their proliferative capacity and promoting cancer progression and metastasis (17), For instance, *Fusobacterium nucleatum* exhibits pro-tumorigenic effects in breast cancer (19). Interestingly, however, intratumoral bacteria (ITB) may also exert a stimulatory effect on immune cell infiltration within tumors, potentially playing a beneficial role in immunotherapy. For instance, in colorectal cancer, studies have found that patients with higher levels of *Fusobacterium* nucleatum exhibit better treatment responses (20), This bacterium appears to modulate TLS via regulatory T cells (21). Conversely, while TLS have been extensively studied in gastrointestinal cancers (22, 23), including HCC (24–26), their mechanisms of formation in HCC specifically remain poorly understood.

In this study, we collected tumor and paired adjacent non-tumor tissues from 153 HCC patients. Hematoxylin and eosin staining and immunofluorescence (IF) were employed to investigate the relationships between ITB, immune cell infiltration, and TLS within the tumor microenvironment (TME). Additionally, we analyzed the clinical relevance of TLS in HCC.

## 2 Methods

This study included a cohort of 153 patients diagnosed with HCC who underwent curative resection at the First Affiliated Hospital of Air Force Medical University between January 2014 and December 2023. All patients were treatment-naïve prior to surgery, and HCC diagnosis was histologically confirmed post-resection. Clinical data and pathological parameters were retrospectively collected from medical records. Patients were followed up every 3 months for the first two years after surgery, and subsequently every 6 months until death or the last follow-up date March 2025。Recurrence-free survival (RFS) was defined as the time from surgery to recurrence or death. Overall survival (OS) was defined as the time from surgery to death or the last follow-up. This study was approved by the Ethics Committee of the First Affiliated Hospital of Air Force Medical University (Approval No. KY20232280-X-1) in accordance with the Declaration of Helsinki. Written informed consent was obtained from all participants.

### 2.1 Hematoxylin and eosin

Human tissue samples were obtained from sterile operating rooms at the First Affiliated Hospital of Air Force Medical University. Fresh liver tumor tissues were immediately transferred into sterile 15 mL conical tubes containing DMEM culture medium. All subsequent sample processing was performed under sterile conditions within a laminar flow hood using autoclaved dissection instruments. Written informed consent was obtained from all patients prior to tissue collection and analysis. All tissue samples were processed into 4-μm-thick formalin-fixed paraffin-embedded sections. Sections were deparaffinized with xylene and rehydrated through a graded ethanol series. After rinsing with deionized water and phosphate-buffered saline (PBS), staining was performed according to the manufacturer’s protocol for the HE staining kit (G1076-500ML, Servicebio), with hematoxylin staining nuclei and eosin staining cytoplasm. Two senior pathologists from the First Affiliated Hospital of Air Force Medical University independently evaluated the HE-stained sections, based on these evaluations, patients were categorized as TLS-positive or TLS-negative. Discrepant cases underwent consensus review by a third senior pathologist.

### 2.2 Multiplex immunofluorescence

Formalin-fixed paraffin-embedded (FFPE) tumor sections from all HCC patients underwent deparaffinization followed by microwave-assisted antigen retrieval. Primary antibodies were incubated at 4°C overnight using the following: Anti-CD20(Abcam, ab78237, 1:50), Anti-LPS(Abcam, ab35654, 1:100), Anti-LTA Antibody(Invitrogen, MA1-7402, 1:50), Anti-CD21(ProteinTech,24374-1-AP,1:200), Anti-CD23(ProteinTech, 60208-1-AP, 1:100), Sections were subsequently incubated with Alexa Fluor 594- and 488-conjugated secondary antibodies (1:200) against mouse and rabbit immunoglobulins for 1 hour at room temperature Nuclei were counterstained with DAPI (G1012, Servicebio), Stained sections were imaged using a fluorescence slide scanner. All fluorescence image quantification and analysis were performed using ImageJ software. This analysis assessed TLS status, as well as the location and expression of intratumoral bacteria, based on patient immunofluorescence staining. To validate the reliability of the antibodies used, we referenced the product datasheets and information provided by the manufacturer (Abcam, Invitrogen), confirming that the antibodies had undergone rigorous validation for immunofluorescence applications. Their specificity was demonstrated by their selective binding to the target LPS epitope. Furthermore, to ensure result reliability, control experiments were included, and repeated PBS washes were performed to minimize non-specific binding, thereby ensuring the accuracy of the LPS staining results.

### 2.3 16S rRNA fluorescence *in situ* hybridization

Tissue sections prepared for fluorescence *in situ* hybridization were fixed and embedded in paraffin. These paraffin-embedded sections were subsequently deparaffinized and rehydrated to water. Antigen/epitope retrieval was performed by boiling the sections in retrieval solution. After thorough washing with phosphate-buffered saline (PBS), sections were incubated in pre-hybridization solution at 37°C for 1 hour. The pre-hybridization solution was then decanted, and hybridization solution containing the EUB338 probe (Servicebio, G3016-3) was applied. Samples were incubated overnight at 37°C in a humidified chamber. Following hybridization, post-hybridization washes were performed. Nuclei were counterstained with DAPI, and images were acquired using a fluorescence microscope. The probe sequence used was EUB338: 5’-CY3-GCT GCC TCC CGT AGG AGT-3’.

### 2.4 Defining TLS and TLS features

Initial TLS localization was performed using HE staining. Subsequent immunofluorescence co-staining with CD20/LPS and CD21/CD23 antibody, Firstly, the expression relationship between CD20 and LPS is illustrated, with CD20 serving as the core structural component of TLS to define both peritumoral TLS and intratumoral TLS. Furthermore, the maturity of TLS is determined by the presence of CD21+ follicular dendritic cells and CD23+ germinal centers. TLS are primarily categorized into: early TLS (Agg), primary follicle-like TLS (FL1), and secondary follicle-like TLS (FL2). Intratumoral TLS are classified as iTLS-, iAgg, iFL1, and iFL2, while peritumoral TLS are classified as pTLS-, pAgg, pFL1, and pFL2.

Furthermore, TME are defined based on the combined distribution of intratumoral and peritumoral TLS, categorized into the following subtypes: “Structured” TME (iTLS+, pTLS+), “Excluded” TME (iTLS-, pFL), “Immature” TME (iTLS-, pAgg), and “Deserted” TME (iTLS-, pTLS-).

## 3 Results

### 3.1 Baseline characteristics

We retrospectively collected 153 HCC patients who underwent surgical resection from the First Affiliated Hospital of Air Force Medical University. Clinicopathological characteristics of these patients are summarized, with a median age of 54 years(range:29-82). The cohort comprised 118 males (77.1%) and 35 females (22.9%), We collected comprehensive clinicopathological data including: hepatitis B infection, liver cirrhosis, smoking history, alcohol consumption history, alpha-fetoprotein (AFP), albumin (Alb), alanine aminotransferase (ALT), neutrophil count, lymphocyte count, monocyte count, TNM stage, tumor number, and differentiation grade. These characteristics are summarized in **Table 1**.

**Table 1:** Clinical, biological, and pathological features of the HCC patients according to the presence of TLS.

Varibles	level	TLS- (n=79)	TLS+ (n=74)	p value		level	TLS- (n=79)	TLS+ (n=74)	p value
Age, No ()					AFP,No ()				
	≤55	43 (28.1%)	40 (26.1%)	0.963		≤300ng/ml	54 (37.3%)	45 (29.4%)	0.329
	>55	36 (23.5%)	34 (22.3%)			>300ng/ml	25 (16.3%)	29 (17%)	
Gender,No ()					SII,No ()				
	male	61 (40.0%)	57 (37.3%)	0.978		≤406.63	60 (39.2%)	51 (33.3%)	0.330
	female	18 (11.8%)	17 (10.9%)			>406.63	19 (12.5%)	23 (15.0%)	
HbsAg,No ()					NLR,No ()				
	with	67 (43.8%)	69 (45.1%)	0.097		≤3.1	63 (41.2%)	52 (34.0%)	0.161
	without	12 (7.8%)	5 (3.3%)			>3.1	16 (7.2%)	22 (17.6%)	
Cirrhosis,No ()					PLR,No ()				
	with	53 (34.6%)	49 (32.0%)	0.996		≤105	49 (32.0%)	38 (24.8%)	0.302
	without	26 (17.0%)	24 (15.7%)			>105	30 (19.6%)	36 (23.6%)	
Alcohol,No ()					LMR,No ()				
	with	11 (7.2%)	12 (7.9%)	0.687		≤2.73	17 (11.1%)	18 (11.8%)	0.838
	without	68 (44.4%)	62 (40.5%)			>2.73	62 (40.5%)	56 (36.6%)	
Smoking,No ()					SIRI,No ()				
	with	19 (12.5%)	21 (13.7%)	0.539		≤1.02	50 (32.7%)	50 (32.7%)	0.999
	without	60 (39.2%)	53 (34.6%)			>1.02	29 (19.0%)	24 (15.6%)	
Tumor number,No ()					PAR,No ()				
	1	66 (43.1%)	65 (42.5%)	0.449		≤3.06	33 (21.6%)	29 (19.0%)	0.776
	>1	13 (8.5%)	9 (5.9%)			>3.06	46 (30.1%)	45 (29.3%)	
TNM stage,No ()					ALRI,No ()				
	I-II	61 (40.0%)	58 (38.0%)	0.862		≤81	69 (45.1%)	68 (44.4%)	0.726
	III- IV	18 (11.8%)	16 (10.2%)			>81	10 (6.5%)	6 (4%)	
Differentiation,No ()					PNI,No ()				
	low-middle	71 (46.4%)	62 (40.5%)	0.263		≤51.15	39 (25.5%)	45 (29.4%)	0.145
	high	8 (5.3%)	12 (7.8%)			>51.15	40 (26.1%)	29 (19.0%)	

Statistical analysis was performed using chi-square tests, AFP, alpha-fetoprotein, SII, Systemic immune-inflammation index, NLR, Neutrophil–lymphocyte ratio, Proplatelet–lymphocyte ratio, LMR, Lymphocyte-to-monocyte ratio, SIRI, systemic inflammation response index, PAR, Platelet-Albumin Ratio, ALRI, AST/Lymphocyte Ratio, PNI, prognostic nutritional index.

### 3.2 Distribution of TLS in HCC

To evaluate the abundance and maturation status of TLS in HCC, we performed hematoxylin and eosin (HE) staining on tumor tissue sections from 153 HCC patients. Tertiary lymphoid structures are defined as CD20+ lymphoid aggregates exceeding 150μm in diameter. TLS identified within the primary tumor are classified as intratumoral TLS detected within 200μm of the tumor-normal parenchyma interface are classified as peritumoral TLS. These structures were characterized by CD20+ B-cell aggregates, confirmed through CD20 immunofluorescence staining in all specimens. According to the described criteria, both intratumoral and peritumoral TLS are classified into two main categories: immature lymphoid aggregates and mature lymphoid follicles, Mature lymphoid follicles are further subdivided into primary follicles and secondary follicles. We classified TLS according to TNM stage and sex, and quantified their distribution across different TLS statuses; these characteristics are summarized in **Table 2**.

**Table 2:** Association of clinical features and number of tertiary lymphoid structures in TME.

TLS number	TNM Stage (n,mean)		P value	Sex (n,mean)		P value
	I-II	III-IV		Male	Female	
Intratumoral Agg	1.403	1.441	0.9125	1.441	1.314	0.7104
Intratumoral FL1	0.370	0.294	0.6045	0.356	0.342	0.9279
Intratumoral FL2	0.050	0.060	0.8640	0.060	0.030	0.5263
Intratumoral TLS	1.824	1.794	0.9507	1.860	1.690	0.7174
Peritumoral Agg	4.010	3.882	0.7719	4.034	3.800	0.5867
Peritumoral FL1	1.290	0.971	0.2336	1.289	0.971	0.2265
Peritumoral FL2	0.261	0.294	0.7238	0.305	0.143	0.0833
Peritumoral TLS	5.555	5.150	0.5156	5.630	4.914	0.2499
Total TLS	7.380	6.941	0.6267	7.483	6.600	0.3201

**Figure 1:**
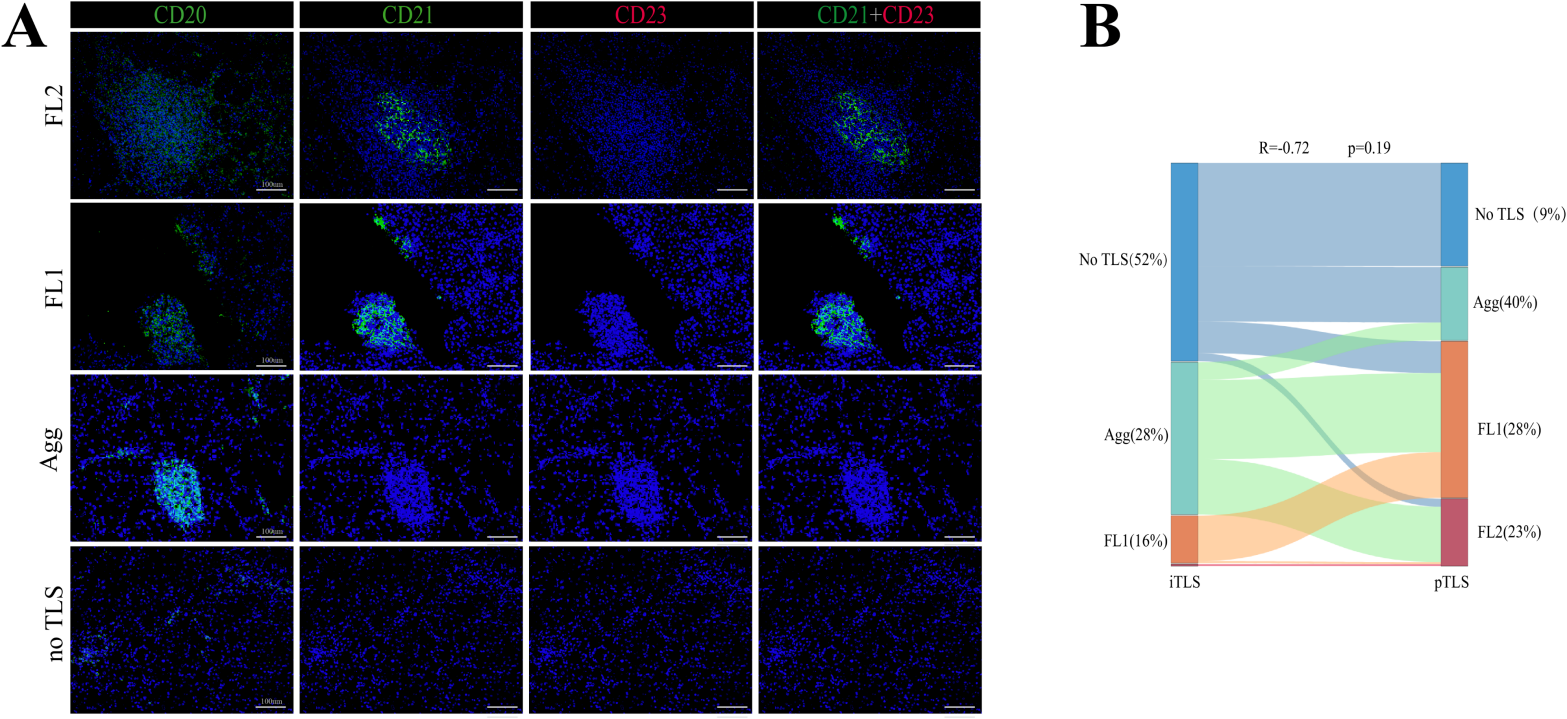
Representative structure of TLSs in HCC. **(A)** Representative images of TLS at different maturity stages within HCC tumors. **(B)** Sankey diagram showing iTLS and pTLS distribution in 153 HCC patients.

**Figure 2:**
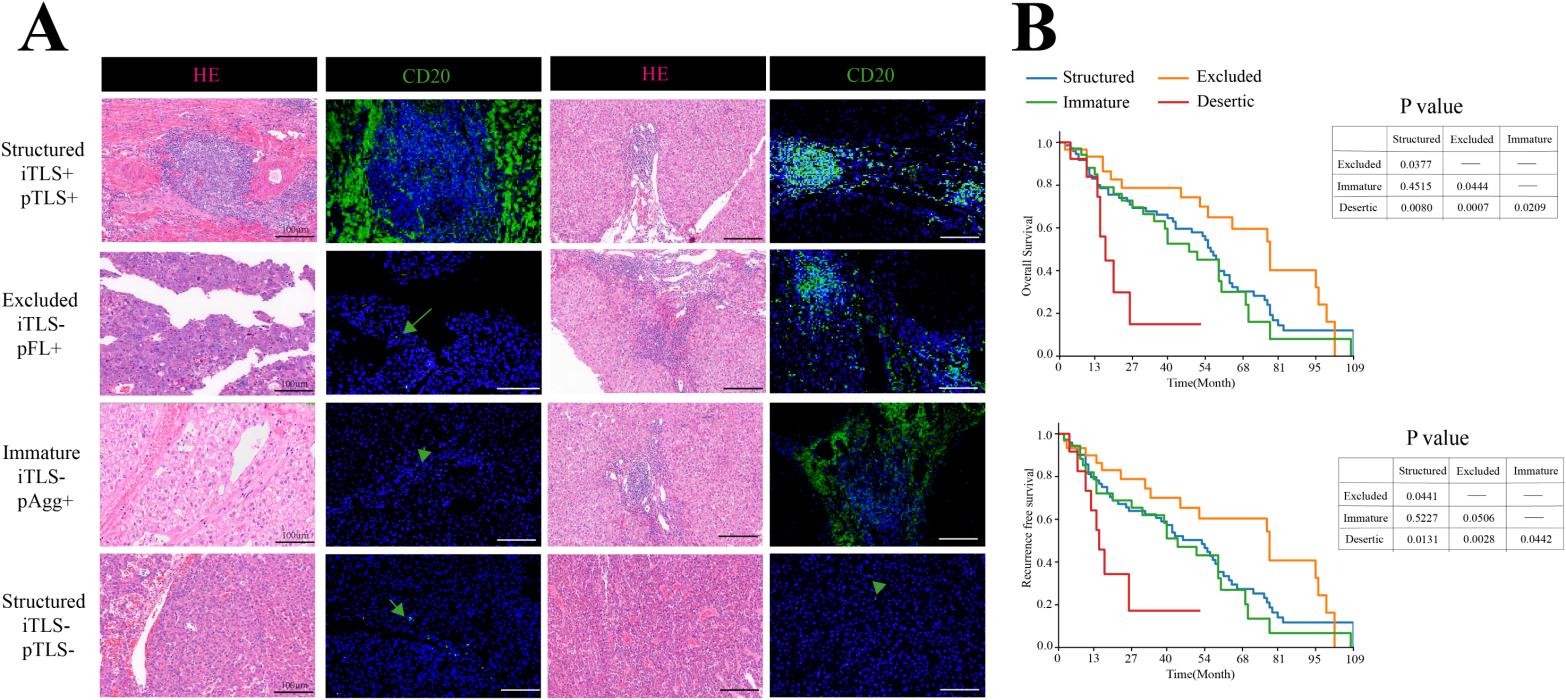
Distribution of TLSs in HCC. **(A)** Representative H&E and IF images of four TME subtypes in HCC. Scale bar, 100mm. **(B)** Kaplan-Meier plots of OS and RFS among four TME stages, n=153.

The numbers of iTLS and pTLS were separately counted for each patient (**Supplementary Table 1**). Analysis revealed no significant correlation between the maturity stages of iTLS and pTLS in HCC (**Figure 1**). Therefore, intra- and peritumoral TLS should be considered as a whole, Based on the maturity and distribution of TLS, the TME of HCC was classified into four categories:”Structured” TME (iTLS+, pTLS+, n=74), “Excluded” TME (iTLS-, pFL+,n=36), “Immature” TME(iTLS-, pAgg+, n=30)And “Deserted” TME (iTLS-, pTLS-,n=13) (**Figure 2A**).

### 3.3 Association between TLS characteristics and survival outcomes (OS/RFS)

**Figure 3:**
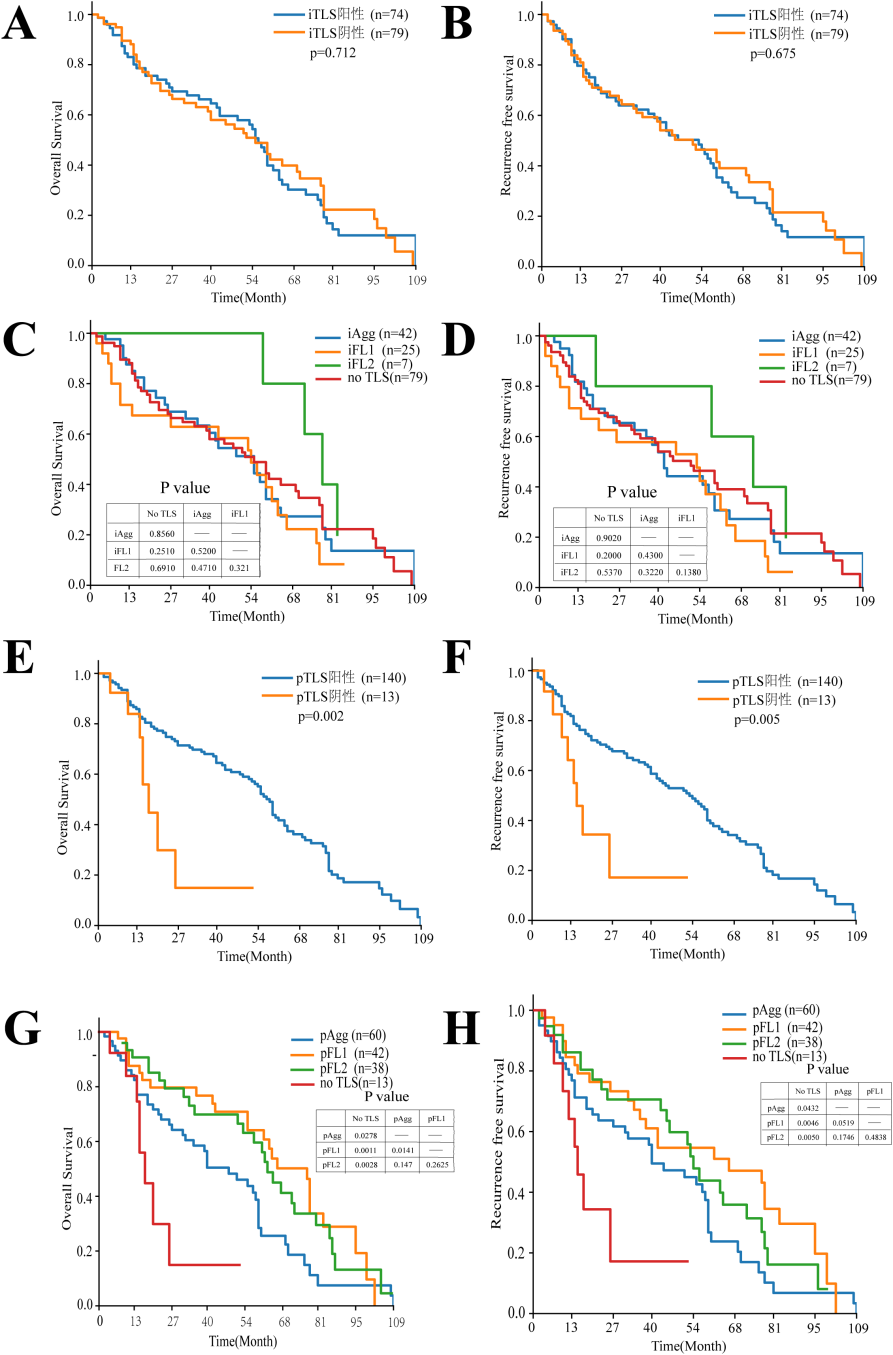
Kaplan-Meier analysis of the relationship between TLS features and OS with RFS in HCC patients. **(A, B)** Kaplan-Meier plots of OS and RFS for iTLS+ and iTLS- patients, n = 153. **(C, D)** Kaplan-Meier plots of OS and RFS showing the survival among iTLSs Stages. **(E, F)** Kaplan-Meier plots of OS and RFS for pTLS+ and pTLS- patients, n = 153. **(G, H)** Kaplan-Meier plots of OS and RFS showing the survival among pTLS Stages.

To investigate the relationship between intra/peritumoral TLS (considered as a whole) and HCC prognosis, Kaplan-Meier curve analysis was performed, This revealed no significant difference in OS or RFS between patients with intratumoral TLS-negative (n=79) and intratumoral TLS-positive (n=74) status, Furthermore, no significant differences in OS or RFS were observed when analyzing groups based on intratumoral TLS maturity: intratumoral Aggregates (n=42), primary follicles (n=25), secondary follicles (n=7), and the no TLS group (n=79). Analysis of peritumoral TLS status, however, showed a significant association. Patients with peritumoral TLS-positive status (n=140) had significantly longer OS (p = 0.002) and RFS (p = 0.005) compared to the peritumoral TLS-negative group (n=13). Further analysis of peritumoral pTLS maturity, compared to the no-TLS group, revealed that patients with pAgg, pFL1, or pFL2 exhibited significantly superior overall survival and recurrence-free survival than those without TLS. In pairwise comparisons among pTLS maturity subtypes, patients with pFL1 demonstrated significantly better OS than those with pAgg, while survival differences between other subtypes did not reach statistical significance. Collectively, these findings indicate that the presence of pTLS is associated with prolonged OS and RFS in HCC patients. Notably, patients with pFL1 showed stronger survival benefits relative to those with pAgg. Analysis of OS and RFS by peritumoral TLS maturity revealed a stronger statistical association for OS compared to RFS (**Figure 3**).

To evaluate the overall prognostic landscape of the hepatocellular carcinoma immune microenvironment, we applied the aforementioned TME classification criteria. Patients with “Structured “TME or “Excluded” TME exhibited significantly longer OS and RFS than those with “Immature” TME or “Deserted” TME. These results demonstrate that a combined assessment of iTLS and pTLS serves as a valuable prognostic biomarker in HCC (**Figure 2B**).

### 3.4 Intratumoral bacteria and CD20+ infiltration association

**Figure 4:**
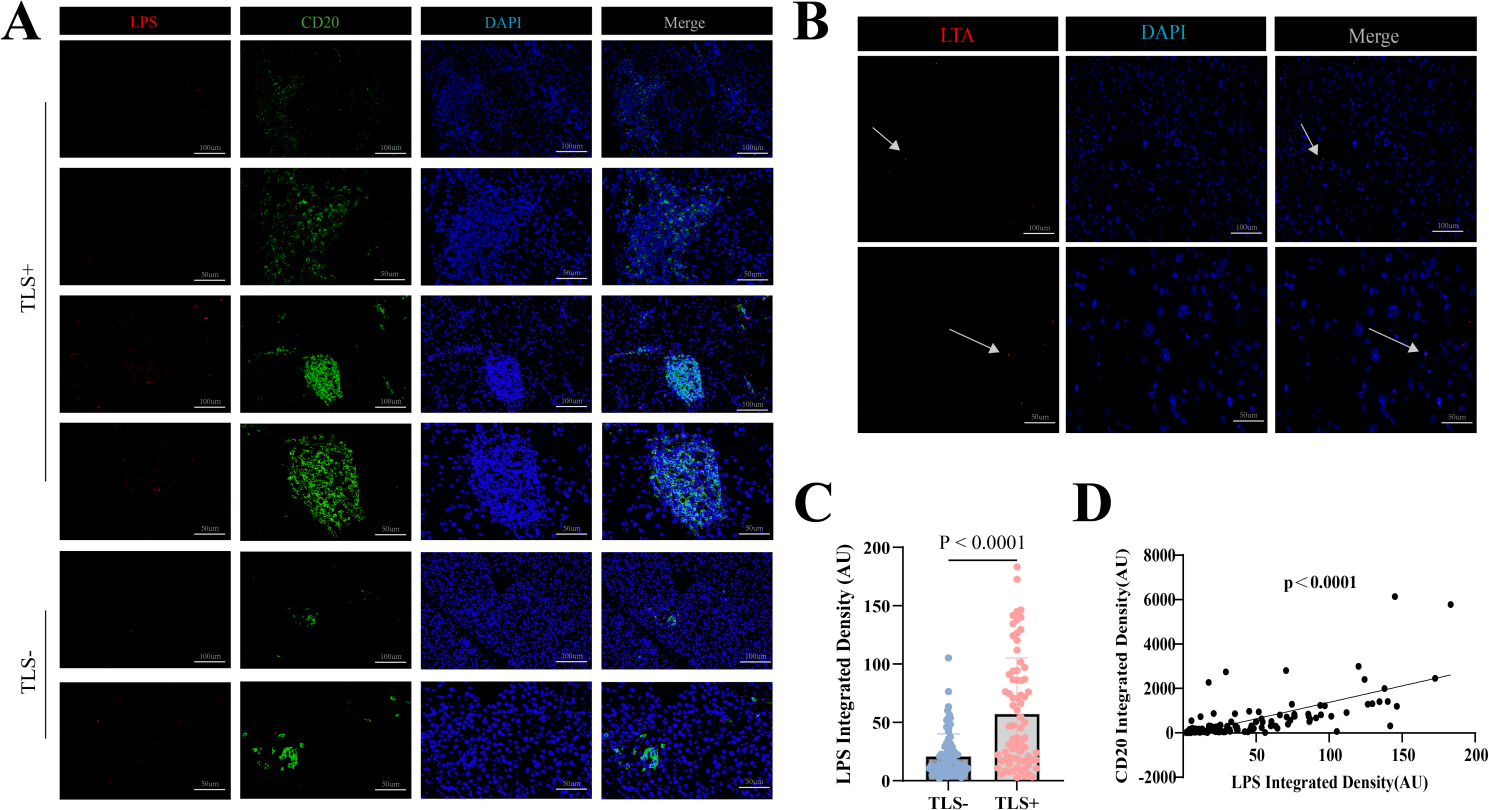
**(A)** Representative IF images of LPS and CD20 within TLS-positive areas. Scale bar, 100mm and 50 mm. **(B)** Representative IF images of LTA. Scale bar, 100mm and 50 mm. **(C)** LPS levels in 153 hepatocellular carcinoma specimens by TLS presence analyzed via IF. **(D)** Correlation analysis of LPS and CD20 levels in 153 hepatocellular carcinoma specimens.

The presence of bacteria in hepatocellular carcinoma (HCC) was confirmed through immunofluorescence (IF) staining and fluorescence *in situ* hybridization (FISH) on patient-derived paraffin-embedded tissue sections. Concurrently, we detected the abundance of lipopolysaccharide (LPS)—a marker of Gram-negative bacteria—and CD20 expression within intratumoral bacteria (**Figure 4A**). Image quantification was performed using ImageJ software, and cutoff values were determined using X-tile software. Subsequent analysis using GraphPad Prism demonstrated a highly significant positive correlation (p < 0.0001) (**Figure 4D**), indicating that higher levels of LPS were associated with increased CD20 expression, And the results demonstrated that both the detection rate and signal intensity of LTA were significantly lower than those of LPS (**Figure 4B**). Furthermore, preoperative blood analysis and biochemical test results were collected from 153 patients. We calculated several systemic inflammatory indices, including the Systemic Immune-Inflammation Index, Neutrophil-to-Lymphocyte Ratio, Lymphocyte-to-Monocyte Ratio, Red cell Distribution Width to Lymphocyte ratio, Systemic Inflammation Response Index, Leukocyte Count Ratio, and Prognostic Nutritional Index. The correlations between these calculated indices and the presence of LPS were then examined. However, the analysis revealed that blood inflammatory markers cannot directly indicate LPS expression levels.

### 3.5 Correlation of intratumoral bacterial load with TLS density and maturity

IF analysis detected ITB, primarily LPS, at varying levels in all 153 cases. The presence of these bacteria may correlate with immune cell expression. Whether intratumoral bacteria confer clinical benefit remains controversial and warrants further investigation.

**Figure 5:**
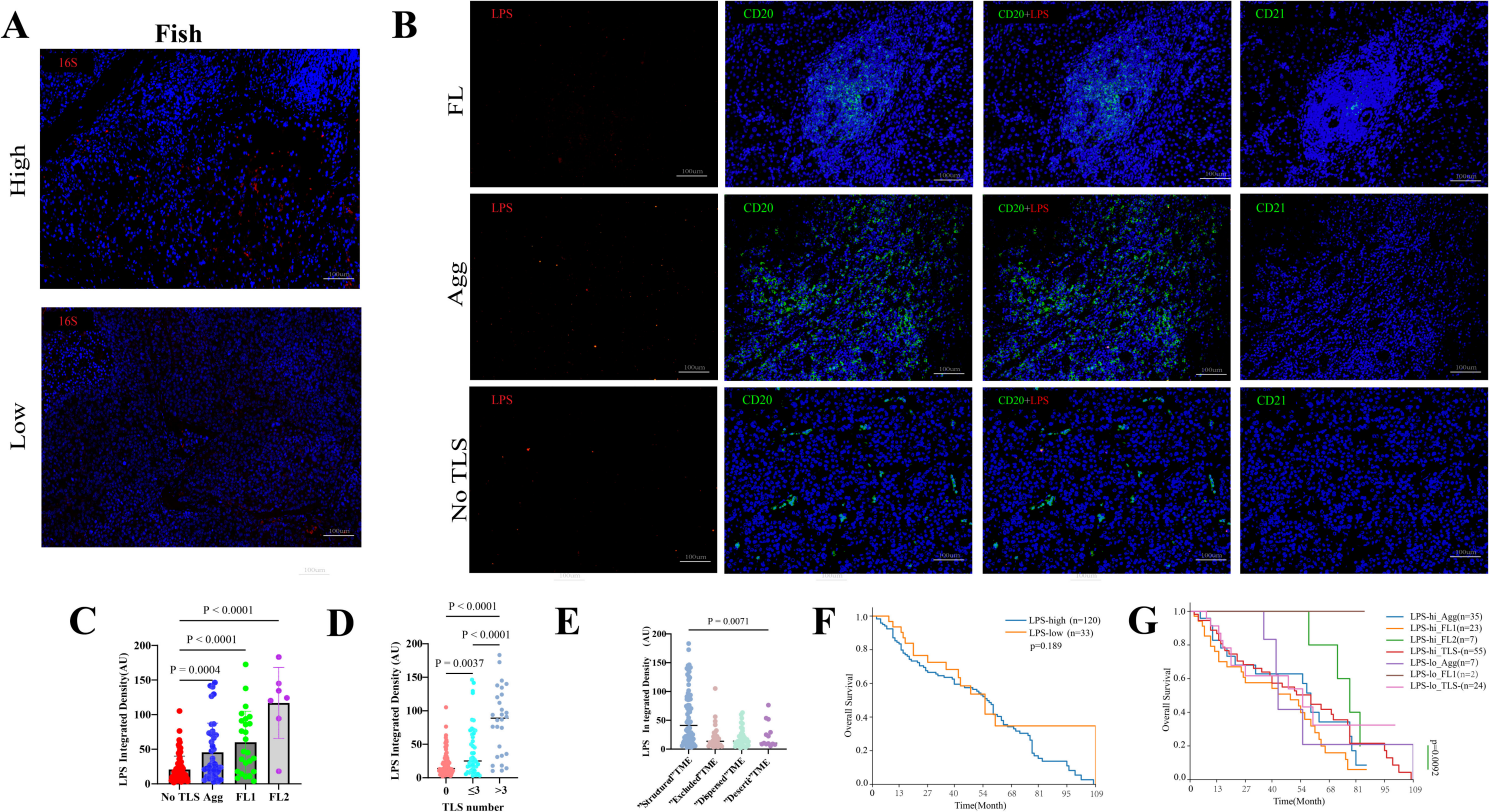
Association between ITB and TLS features (abundance, maturity) in HCC. **(A)** Representative confocal micrographs of 16S rRNA FISH in HCC tissue. Cellular nuclei are labeled in blue, and bacteria in red. Scale bars, 100mm **(B)** Representative images of LPS, CD20, and CD21 across TLS maturity stages, Scale bar, 100mm. **(C)** LPS levels by TLS maturity in 153 HCC specimens analyzed via IF. **(D)** LPS levels by TLS numerical abundance in 153 HCC specimens analyzed via IF. **(E)** LPS levels by TME subtype in 153 HCC specimens analyzed via IF. **(F)** Kaplan-Meier plots by LPS group. **(G)** Kaplan-Meier plots by combined LPS/TLS groups.

Our study found that higher levels of intratumoral bacteria (ITB) correlated with increased CD20 expression, To further investigate the association between ITB and TLS, we further investigated the relationship between ITB and TLS (**Figures 5A, B**). We quantified TLS counts and ITB load in patient samples. Using paraffin-embedded sections, HE staining and IF assessed LPS levels and evaluated their association with intratumoral TLS. Patients with TLS showed significantly elevated ITB levels compared to those without TLS (**Figure 4C**). When patients were stratified by iTLS count (0, ≤3, >3), higher LPS expression correlated with increased TLS numbers (**Figure 5D**). Analysis incorporating TLS maturation status revealed that higher maturity levels were associated with significantly increased ITB load, indicating a positive correlation (**Figure 5C**). Comparing structured TME versus desert TME states revealed a significant difference in ITB expression (p=0.0071) (**Figure 5E**). Finally, ITB load alone showed no significant correlation with patient prognosis. the combined analysis of ITB and TLS status revealed that patients with low bacterial load and TLS-negative status had the worst prognosis, while those with high bacterial load and FL2-positive TLS status showed relatively better outcomes (**Figures 5F, G**).

## 4 Discussion

In recent years, the intratumoral microbiota, particularly bacteria, is increasingly recognized as a key regulator of tumor progression and responses to immunotherapy. Previous studies have suggested that ITB may influence the tumor microenvironment and immunotherapy efficacy by inducing local necrosis, suppressing T-cell infiltration, or modulating immune checkpoint pathways (18, 27–29). Notably, these microbes may exhibit dual roles: while potentially promoting cancer progression, they can also exert antitumor effects through immune cell recruitment (30). In hepatocellular carcinoma, the presence and functional significance of ITB remain to be fully explored. Our study analyzed 153 retrospectively collected HCC surgical resection samples. Using 16S rRNA fluorescence *in situ* hybridization (FISH) and immunofluorescence staining for lipopolysaccharide and lipoteichoic acid, we observed bacteria exhibiting a punctate pattern within the perinuclear region of tumor cells. These bacteria were predominantly Gram-negative, although their abundance was lower than in other gastrointestinal tumors. Gram-negative bacteria were primarily localized within tumor cells and stromal cell populations, with CD20+ B cells also positioned near LPS signals. These findings suggest that ITB may exist primarily intracellularly, within both cancer and immune cells. This observation aligns with the findings of Xue et al., who reported distinct bacterial communities between HCC tumor and non-tumor tissues, with some bacteria correlating with metabolites (31). It also resonates with Liu et al.’s observation of bacterial presence within inflammatory areas alongside CD8+ T cells and myeloid-derived suppressor cells in HBV-related HCC (32). Collectively, these findings suggest that ITB in HCC may possess the potential to modulate the local tumor microenvironment, representing a promising therapeutic target worthy of further exploration (33).

Given the importance of immunotherapy in cancer, we focused on the impact of ITB on the tumor microenvironment, particularly tertiary lymphoid structures (TLS). As key immune components within the TME, TLS have been associated with favorable prognoses across multiple cancers, including melanoma (34), colon cancer (35), lung cancer (36), pancreatic cancer (22), and esophageal cancer (37). However, the prognostic significance of TLS in HCC—encompassing both intratumoral TLS and peritumoral TLS—remains controversial. Emerging research indicates that immunotherapy modulates TLS density, and higher intratumoral TLS levels correlate with improved recurrence-free survival (24). To better evaluate the overall relevance of TLS in HCC, we adopted a novel approach: rather than assessing iTLS or pTLS independently (as in prior studies), we analyzed them as an integrated TME immune structure for prognostic evaluation. A key finding of this study is the significant correlation between enrichment of Gram-negative bacteria in HCC tissues and TLS presence, whereas Gram-positive bacteria were relatively scarce. Notably, our immunofluorescence (IF) analysis revealed a spatial association between CD20+ B cells (core components of TLS) and bacterial localization. This suggests ITB, particularly Gram-negative species, may promote TLS formation and maturation by recruiting CD20+ B cells.

This finding is highly innovative, representing the first direct *in situ* linkage of intratumorally colonizing bacteria to TLS establishment in a solid tumor, specifically HCC. It extends previous observations that gut microbiota may influence HCC TLS (38), underscoring the importance of studying the *local* tumor microbiota—which may exert more direct effects within the TME compared to gut microbes. Furthermore, while Sun et al. found intratumoral microbes remodel the HCC TME and influence CD68+ macrophage infiltration (39), our study specifically focuses on the TLS niche. We identify CD20+ B cells as potential mediators of bacterial effects, promoting TLS quantity and maturation. This provides a new perspective on how microbes precisely regulate the HCC immune landscape. Nevertheless, a fundamental and clinically significant question remains: How do intratumoral bacteria specifically drive CD20+ B cell recruitment and subsequent TLS formation/maturation?

Based on our findings, we hypothesize that bacterial-derived antigens may activate intratumoral B cells, promoting their expansion and facilitating TLS construction and maintenance. The interaction between B cells and T cells—mediated through mechanisms such as antigen presentation and co-stimulatory molecules—is crucial for TLS maturation and function (8). Our observation that bacteria predominantly localize to the perinuclear region could enhance the accessibility of their products to immune cells. Furthermore, bacterial metabolites, as mentioned by Xue et al., may also contribute to regulating immune cell migration and function (31). B-cell-specific chemokines, such as CXCL13, are key drivers for recruiting B cells to inflammatory sites and initiating TLS formation (23, 40). Therefore, given that LPS is a potent Toll-like receptor 4 agonist, we speculate that ITB might stimulate cells within the TME, via receptor signaling pathways like Toll-like receptor 4, to produce critical chemokines such as CXCL13 (41, 42). This could directly recruit circulating CD20+ B cells to the tumor site, thereby promoting TLS formation. An intriguing parallel observation comes from clear cell renal cell carcinoma, where intratumoral fungi were found to suppress lipid catabolism and induce T cell exhaustion (43). This further highlights the functional diversity of the tumor microbiome. Consequently, a more comprehensive understanding of immune cell-mediated mechanisms shaping microbial distribution within tumors requires consideration of other components of the tumor microbiome, including fungal species. While gut microbes may serve as a source of intratumoral bacteria – primarily translocating via the enterohepatic circulation – studies in pancreatic cancer report no such association (44). These mechanisms represent plausible pathways, future studies employing metagenomic sequencing and strain-specific culture are essential for validation.

This study has several limitations. Our characterization of bacteria was limited; we used LPS as a pan-marker for Gram-negative bacteria and were unable to identify specific bacterial species or strains. Furthermore, LTA staining yielded a low positive rate and weak signals in our study. While this does not preclude a role for Gram-positive bacteria in localized microenvironments, our current data indicate their overall contribution to TLS formation is likely limited. Future validation using metagenomic sequencing and strain-specific culture is essential and represents a primary limitation of this work. Importantly, the immunomodulatory functions of different bacterial species can vary significantly, even exhibiting opposing effects. Consequently, the observed association between intratumoral bacteria and TLS in HCC may be primarily driven by immunogenic Gram-negative species. Future research should employ 16S rRNA gene sequencing and single-bacterium isolation/culture techniques to precisely identify key bacterial species associated with TLS formation in HCC and characterize their functional profiles. Additionally, our retrospective cohort had a limited sample size and was from a single center. HCC etiology, geographic region, and patient lifestyle factors could significantly influence intratumoral microbiota composition and the TME. Larger-scale, multi-center prospective studies with detailed epidemiological data are needed to validate and generalize our findings and explore heterogeneity across different patient subgroups.

In summary, this study reveals a significant correlation between the enrichment of intratumoral Gram-negative bacteria and the presence of TLS in HCC. It is the first to propose a mechanism whereby bacteria, potentially via recruitment of CD20+ B cells, may promote TLS formation and maturation, providing a novel perspective on the regulation of the HCC immune microenvironment. We established an integrated iTLS/pTLS evaluation standard for stratifying HCC patients and predicting prognosis based on TLS. Despite limitations such as the lack of precise bacterial classification, this work provides a foundation for future research to identify key pro-TLS bacterial species and elucidate their mechanisms of action. Such efforts promise not only to deepen our understanding of TLS biology but also to pave the way for novel therapeutic strategies. Combining interventions targeting these pathways with existing immunotherapies, such as immune checkpoint inhibitors (ICIs), holds potential to overcome immune resistance and ultimately improve the long-term clinical outcomes of HCC patients. Continued exploration of the intratumoral microbiota – including bacteria, fungi, and other components – and their interactions with host immunity represents a highly promising new direction in cancer immunotherapy.

## Data Availability

The original contributions presented in the study are included in the article/**Supplementary Material**. Further inquiries can be directed to the corresponding author.
