# Supplementary material for: Intratumoral bacterial load and tertiary lymphoid structure density in hepatocellular carcinoma: association and prognostic significance
Source: Front Immunol. 2025 Sep 2;16:1652433. doi: 10.3389/fimmu.2025.1652433 (PMC12436420; doi:10.3389/fimmu.2025.1652433)
Supplement: Supplementary file 1 [file Table1.docx]

Supplementary Material

# Supplementary Figures and Tables

**Supplementary Table1：**Distribution of Different Types of Tertiary Lymphoid Structures in 153 Hepatocellular Carcinoma Patients

| **Patient ID** | **intratumoral Agg Number** | **intratumoral Agg Number** | **intratumoral FL2 Number** | **Total intratumoral TLS Number** | **peritumoral Agg Number** | **peritumoral FL1 Number** | **peritumoral FL2 Number** | **Total peritumoral TLS Number** | **Total TLS Number** |
| --- | --- | --- | --- | --- | --- | --- | --- | --- | --- |
| E68463 | 2 | 3 | 0 | 5 | 3 | 3 | 0 | 6 | 8 |
| D76384 | 0 | 1 | 0 | 1 | 2 | 2 | 0 | 4 | 4 |
| D69295 | 0 | 1 | 0 | 1 | 6 | 2 | 0 | 8 | 8 |
| E13095 | 0 | 1 | 0 | 1 | 5 | 2 | 1 | 8 | 8 |
| E12913 | 0 | 1 | 0 | 1 | 6 | 2 | 1 | 9 | 9 |
| D78651 | 2 | 3 | 0 | 5 | 4 | 0 | 0 | 4 | 8 |
| E95103 | 8 | 9 | 2 | 19 | 4 | 2 | 1 | 7 | 19 |
| E87389 | 3 | 4 | 0 | 7 | 4 | 1 | 0 | 5 | 10 |
| D61964 | 3 | 4 | 0 | 7 | 3 | 0 | 0 | 3 | 6 |
| D81935 | 0 | 1 | 0 | 1 | 0 | 0 | 0 | 0 | 0 |
| E12378 | 2 | 3 | 0 | 5 | 4 | 3 | 1 | 8 | 10 |
| E84440 | 0 | 1 | 0 | 1 | 4 | 3 | 1 | 8 | 8 |
| E99152 | 0 | 1 | 0 | 1 | 3 | 0 | 0 | 3 | 3 |
| E94533 | 3 | 4 | 0 | 7 | 6 | 4 | 1 | 11 | 16 |
| D70306 | 2 | 3 | 0 | 5 | 8 | 4 | 1 | 13 | 17 |
| E83603 | 2 | 3 | 0 | 5 | 8 | 4 | 0 | 12 | 15 |
| A18687 | 0 | 1 | 0 | 1 | 4 | 4 | 0 | 8 | 8 |
| D67818 | 0 | 1 | 0 | 1 | 0 | 0 | 0 | 0 | 0 |
| D53487 | 0 | 1 | 0 | 1 | 3 | 0 | 0 | 3 | 3 |
| E95883 | 2 | 3 | 1 | 6 | 8 | 2 | 0 | 10 | 16 |
| D76710 | 0 | 1 | 0 | 1 | 0 | 0 | 0 | 0 | 0 |
| D27019 | 0 | 1 | 0 | 1 | 6 | 0 | 0 | 6 | 6 |
| D22139 | 2 | 3 | 0 | 5 | 5 | 0 | 0 | 5 | 9 |
| D29362 | 0 | 1 | 0 | 1 | 5 | 0 | 0 | 5 | 5 |
| D27752 | 0 | 1 | 0 | 1 | 3 | 0 | 0 | 3 | 3 |
| D17098 | 2 | 3 | 0 | 5 | 4 | 0 | 0 | 4 | 6 |
| D53617 | 0 | 1 | 0 | 1 | 3 | 0 | 0 | 3 | 3 |
| D81787 | 3 | 4 | 0 | 7 | 6 | 0 | 0 | 6 | 9 |
| D54938 | 0 | 1 | 0 | 1 | 6 | 2 | 0 | 8 | 8 |
| D41897 | 2 | 3 | 0 | 5 | 10 | 2 | 0 | 12 | 14 |
| D77382 | 0 | 1 | 0 | 1 | 6 | 0 | 0 | 6 | 6 |
| F21128 | 5 | 6 | 1 | 12 | 8 | 1 | 0 | 9 | 18 |
| F32527 | 0 | 1 | 0 | 1 | 5 | 2 | 0 | 7 | 7 |
| F15330 | 0 | 1 | 0 | 1 | 2 | 0 | 0 | 2 | 2 |
| F17233 | 4 | 5 | 1 | 10 | 4 | 3 | 0 | 7 | 14 |
| F07750 | 3 | 4 | 0 | 7 | 4 | 0 | 0 | 4 | 8 |
| F36863 | 0 | 1 | 0 | 1 | 6 | 0 | 0 | 6 | 6 |
| F38836 | 4 | 5 | 0 | 9 | 4 | 2 | 1 | 7 | 11 |
| F37690 | 6 | 7 | 0 | 13 | 5 | 0 | 0 | 5 | 14 |
| F25995 | 0 | 1 | 0 | 1 | 2 | 0 | 0 | 2 | 2 |
| F31058 | 0 | 1 | 0 | 1 | 5 | 0 | 0 | 5 | 5 |
| F24305 | 4 | 5 | 0 | 9 | 8 | 2 | 0 | 10 | 14 |
| F10373 | 3 | 4 | 0 | 7 | 6 | 3 | 1 | 10 | 13 |
| F35843 | 2 | 3 | 0 | 5 | 3 | 2 | 1 | 6 | 9 |
| F28624 | 2 | 3 | 0 | 5 | 8 | 4 | 1 | 13 | 15 |
| F28152 | 0 | 1 | 0 | 1 | 5 | 2 | 0 | 7 | 7 |
| F30005 | 0 | 1 | 0 | 1 | 6 | 4 | 0 | 10 | 10 |
| F08594 | 2 | 3 | 0 | 5 | 3 | 1 | 0 | 4 | 6 |
| F02261 | 4 | 5 | 0 | 9 | 4 | 3 | 2 | 9 | 13 |
| F17184 | 3 | 4 | 0 | 7 | 4 | 1 | 1 | 6 | 9 |
| F28268 | 4 | 5 | 0 | 9 | 6 | 1 | 1 | 8 | 13 |
| F07573 | 6 | 7 | 0 | 13 | 7 | 2 | 2 | 11 | 19 |
| F29431 | 3 | 4 | 0 | 7 | 5 | 0 | 0 | 5 | 8 |
| F08972 | 2 | 3 | 0 | 5 | 8 | 3 | 0 | 11 | 13 |
| F31322 | 0 | 1 | 0 | 1 | 6 | 2 | 0 | 8 | 8 |
| F22420 | 3 | 4 | 0 | 7 | 3 | 2 | 0 | 5 | 8 |
| F32111 | 8 | 9 | 1 | 18 | 5 | 0 | 0 | 5 | 16 |
| F03875 | 0 | 1 | 0 | 1 | 4 | 1 | 0 | 5 | 5 |
| F16440 | 3 | 4 | 0 | 7 | 3 | 2 | 0 | 5 | 10 |
| E78743 | 2 | 3 | 0 | 5 | 4 | 3 | 0 | 7 | 9 |
| E77612 | 1 | 2 | 0 | 3 | 3 | 0 | 0 | 3 | 4 |
| F21413 | 0 | 1 | 0 | 1 | 6 | 2 | 0 | 8 | 8 |
| F15963 | 2 | 3 | 0 | 5 | 8 | 2 | 0 | 10 | 13 |
| F11042 | 0 | 1 | 0 | 1 | 3 | 3 | 1 | 7 | 7 |
| E74869 | 3 | 4 | 0 | 7 | 6 | 2 | 0 | 8 | 11 |
| F58107 | 2 | 3 | 0 | 5 | 6 | 2 | 1 | 9 | 13 |
| G20631 | 0 | 1 | 0 | 1 | 4 | 0 | 0 | 4 | 4 |
| G21662 | 0 | 1 | 0 | 1 | 5 | 0 | 0 | 5 | 5 |
| G24419 | 0 | 1 | 0 | 1 | 2 | 0 | 0 | 2 | 2 |
| G21467 | 4 | 5 | 0 | 9 | 5 | 2 | 1 | 8 | 14 |
| G19888 | 0 | 1 | 0 | 1 | 3 | 0 | 0 | 3 | 3 |
| G19980 | 0 | 1 | 0 | 1 | 2 | 0 | 0 | 2 | 2 |
| G24505 | 1 | 2 | 0 | 3 | 3 | 1 | 0 | 4 | 5 |
| G20682 | 2 | 3 | 0 | 5 | 5 | 0 | 0 | 5 | 8 |
| G20526 | 0 | 1 | 0 | 1 | 2 | 0 | 0 | 2 | 2 |
| G14734 | 1 | 2 | 0 | 3 | 8 | 2 | 1 | 11 | 13 |
| G19060 | 1 | 2 | 0 | 3 | 6 | 0 | 0 | 6 | 7 |
| G25096 | 0 | 1 | 0 | 1 | 5 | 0 | 0 | 5 | 5 |
| G21028 | 3 | 4 | 0 | 7 | 4 | 0 | 0 | 4 | 7 |
| G25172 | 3 | 4 | 0 | 7 | 4 | 0 | 0 | 4 | 8 |
| F56676 | 1 | 2 | 0 | 3 | 3 | 3 | 1 | 7 | 9 |
| F95387 | 4 | 5 | 0 | 9 | 6 | 2 | 1 | 9 | 13 |
| F97218 | 4 | 5 | 0 | 9 | 5 | 4 | 2 | 11 | 16 |
| E07113 | 0 | 1 | 0 | 1 | 2 | 2 | 1 | 5 | 5 |
| G50877 | 5 | 6 | 0 | 11 | 8 | 1 | 1 | 10 | 15 |
| D61662 | 3 | 4 | 0 | 7 | 4 | 2 | 0 | 6 | 9 |
| D42695 | 0 | 1 | 0 | 1 | 6 | 1 | 1 | 8 | 8 |
| C26566 | 3 | 4 | 1 | 8 | 1 | 4 | 1 | 6 | 11 |
| D91432 | 0 | 1 | 0 | 1 | 6 | 3 | 0 | 9 | 9 |
| D29856 | 0 | 1 | 0 | 1 | 6 | 2 | 0 | 8 | 8 |
| D30450 | 0 | 1 | 0 | 1 | 3 | 0 | 0 | 3 | 3 |
| D23795 | 2 | 3 | 0 | 5 | 4 | 0 | 0 | 4 | 6 |
| D29990 | 0 | 1 | 0 | 1 | 7 | 0 | 0 | 7 | 7 |
| F18228 | 0 | 1 | 0 | 1 | 6 | 1 | 1 | 8 | 8 |
| F04607 | 0 | 1 | 0 | 1 | 8 | 2 | 0 | 10 | 10 |
| I62079 | 0 | 1 | 0 | 1 | 0 | 0 | 0 | 0 | 0 |
| I52091 | 1 | 2 | 0 | 3 | 2 | 2 | 0 | 4 | 5 |
| I36073 | 0 | 1 | 0 | 1 | 2 | 0 | 0 | 2 | 2 |
| J04514 | 2 | 3 | 0 | 5 | 3 | 1 | 0 | 4 | 7 |
| I32013 | 0 | 1 | 0 | 1 | 1 | 0 | 0 | 1 | 1 |
| I68113 | 0 | 1 | 0 | 1 | 0 | 0 | 0 | 0 | 0 |
| J05843 | 0 | 1 | 0 | 1 | 0 | 0 | 0 | 0 | 0 |
| I46662 | 3 | 4 | 0 | 7 | 2 | 0 | 0 | 2 | 5 |
| J03533 | 0 | 1 | 0 | 1 | 2 | 0 | 0 | 2 | 2 |
| I66790 | 0 | 1 | 0 | 1 | 6 | 4 | 1 | 11 | 11 |
| I63588 | 0 | 1 | 0 | 1 | 8 | 2 | 0 | 10 | 10 |
| J05777 | 0 | 1 | 0 | 1 | 4 | 1 | 0 | 5 | 5 |
| D66748 | 3 | 4 | 0 | 7 | 5 | 0 | 0 | 5 | 8 |
| D87008 | 0 | 1 | 0 | 1 | 0 | 0 | 0 | 0 | 0 |
| E29204 | 0 | 1 | 0 | 1 | 3 | 0 | 0 | 3 | 3 |
| D86751 | 4 | 5 | 0 | 9 | 2 | 2 | 1 | 5 | 11 |
| D22226 | 2 | 3 | 0 | 5 | 3 | 1 | 0 | 4 | 6 |
| D76384 | 0 | 1 | 0 | 1 | 7 | 4 | 1 | 12 | 12 |
| D06370 | 3 | 4 | 0 | 7 | 6 | 2 | 1 | 9 | 12 |
| E61945 | 0 | 1 | 0 | 1 | 3 | 0 | 0 | 3 | 3 |
| D36332 | 0 | 1 | 0 | 1 | 4 | 0 | 0 | 4 | 4 |
| D68133 | 2 | 3 | 0 | 5 | 6 | 0 | 0 | 6 | 10 |
| D99854 | 0 | 1 | 0 | 1 | 5 | 0 | 0 | 5 | 5 |
| D68024 | 6 | 7 | 1 | 14 | 6 | 0 | 0 | 6 | 15 |
| D52636 | 0 | 1 | 0 | 1 | 0 | 0 | 0 | 0 | 0 |
| D09846 | 0 | 1 | 0 | 1 | 0 | 0 | 0 | 0 | 0 |
| D15183 | 0 | 1 | 0 | 1 | 6 | 0 | 0 | 6 | 6 |
| D22139 | 3 | 4 | 0 | 7 | 4 | 2 | 1 | 7 | 10 |
| D36945 | 0 | 1 | 0 | 1 | 2 | 0 | 0 | 2 | 2 |
| D40667 | 3 | 4 | 0 | 7 | 2 | 0 | 0 | 2 | 5 |
| D43297 | 2 | 3 | 0 | 5 | 2 | 2 | 0 | 4 | 6 |
| E13463 | 0 | 1 | 0 | 1 | 0 | 0 | 0 | 0 | 0 |
| D49028 | 0 | 1 | 0 | 1 | 0 | 0 | 0 | 0 | 0 |
| D49562 | 0 | 1 | 0 | 1 | 3 | 0 | 0 | 3 | 3 |
| E21450 | 0 | 1 | 0 | 1 | 4 | 0 | 0 | 4 | 4 |
| E26582 | 2 | 3 | 0 | 5 | 3 | 2 | 0 | 5 | 8 |
| E06853 | 0 | 1 | 0 | 1 | 0 | 0 | 0 | 0 | 0 |
| E26586 | 0 | 1 | 0 | 1 | 2 | 0 | 0 | 2 | 2 |
| G39307 | 4 | 5 | 0 | 9 | 3 | 1 | 0 | 4 | 10 |
| G58566 | 0 | 1 | 0 | 1 | 1 | 1 | 0 | 2 | 2 |
| G56136 | 0 | 1 | 0 | 1 | 1 | 3 | 0 | 4 | 4 |
| G41185 | 3 | 4 | 0 | 7 | 6 | 0 | 0 | 6 | 9 |
| G43019 | 0 | 1 | 0 | 1 | 2 | 4 | 0 | 6 | 6 |
| G28414 | 0 | 1 | 0 | 1 | 2 | 0 | 0 | 2 | 2 |
| G54911 | 0 | 1 | 0 | 1 | 6 | 2 | 1 | 9 | 9 |
| F11411 | 2 | 3 | 0 | 5 | 2 | 1 | 1 | 4 | 6 |
| G53358 | 0 | 1 | 0 | 1 | 3 | 0 | 0 | 3 | 3 |
| G56603 | 0 | 1 | 0 | 1 | 0 | 0 | 0 | 0 | 0 |
| G28413 | 2 | 3 | 0 | 5 | 4 | 0 | 0 | 4 | 6 |
| G58094 | 3 | 4 | 0 | 7 | 2 | 4 | 1 | 7 | 10 |
| G63659 | 0 | 1 | 0 | 1 | 2 | 0 | 0 | 2 | 2 |
| G63609 | 0 | 1 | 0 | 1 | 4 | 4 | 1 | 9 | 9 |
| G40493 | 3 | 4 | 0 | 7 | 5 | 4 | 1 | 10 | 13 |
| G63996 | 0 | 1 | 0 | 1 | 3 | 0 | 0 | 3 | 3 |
| G37932 | 2 | 3 | 0 | 5 | 2 | 3 | 1 | 6 | 8 |
| G31568 | 3 | 4 | 0 | 7 | 2 | 0 | 0 | 2 | 5 |
| G42995 | 0 | 1 | 0 | 1 | 2 | 2 | 0 | 4 | 4 |
| G34513 | 2 | 3 | 0 | 5 | 2 | 1 | 0 | 3 | 5 |

**Supplementary Figure 1.** Kaplan-Meier analysis of the relationship between TLS features and OS with RFS in HCC Patients
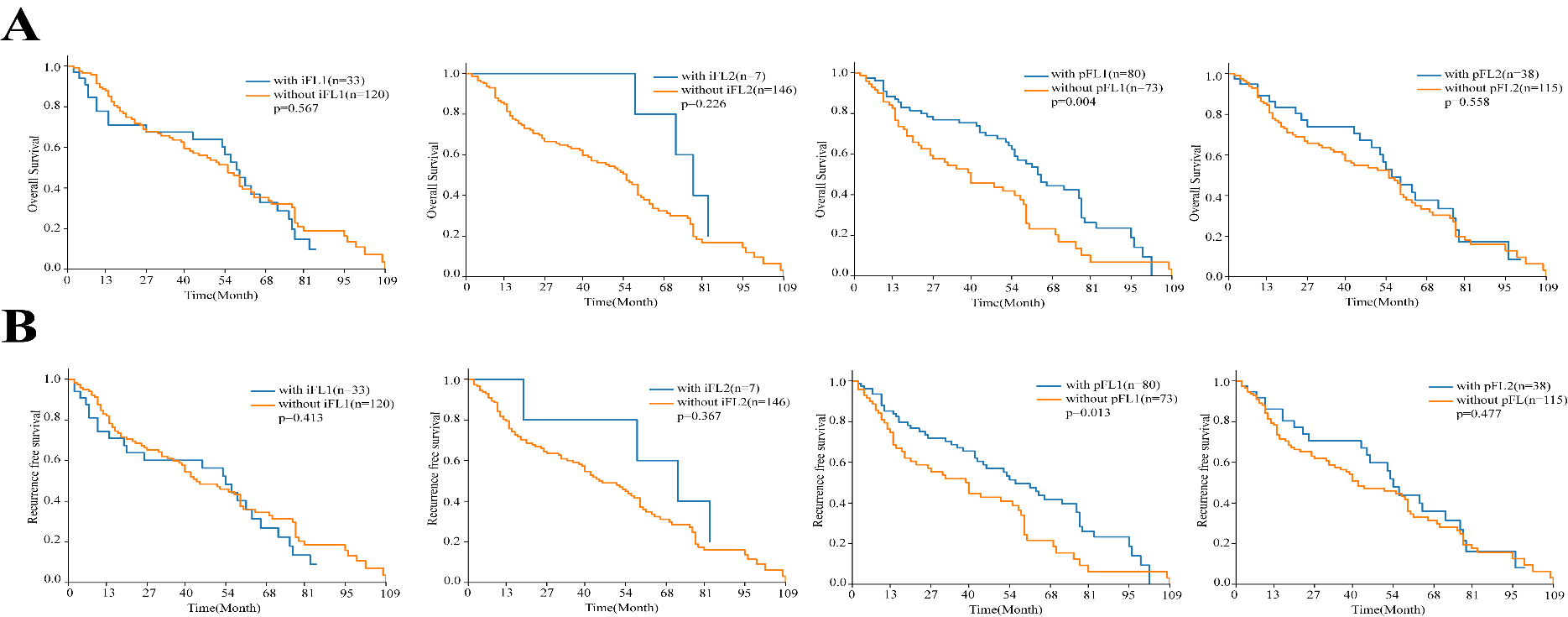


A: Kaplan-Meier plots of OS for patients, n = 153.

B: Kaplan-Meier plots of RFS for patients, n = 153.
